# Retrospective Multicenter Long-Term Follow-up Analysis of Prognostic Risk Factors for Recurrence-Free, Metastasis-Free, Cancer-Specific, and Overall Survival After Curative Nephrectomy in Non-metastatic Renal Cell Carcinoma

**DOI:** 10.3389/fonc.2019.00859

**Published:** 2019-09-04

**Authors:** Sung Han Kim, Boram Park, Eu Chang Hwang, Sung-Hoo Hong, Chang Wook Jeong, Cheol Kwak, Seok Soo Byun, Jinsoo Chung

**Affiliations:** ^1^Department of Urology, Urologic Cancer Center, Research Institute and Hospital of National Cancer Center, Goyang-si, South Korea; ^2^Biostatistics Collaboration Team, Research Core Center, National Cancer Center, Research Institute, Goyang-si, South Korea; ^3^Department of Urology, Chonnam National University Medical School, Gwangju, South Korea; ^4^Department of Urology, Seoul St. Mary's Hospital, The Catholic University of Korea, Seoul, South Korea; ^5^Department of Urology, Seoul National University Hospital, Seoul, South Korea; ^6^Department of Urology, Seoul National Medical University and Bundang Hospital, Seongnam-si, South Korea

**Keywords:** metastasis, prognosis, non-metastatic, renal cell carcinoma, nephrectomy, survival, risk factor

## Abstract

We evaluated prognostic risk factors of recurrence-free survival (RFS), metastasis-free survival (MFS), cancer-specific survival (CSS), and overall survival (OS) outcomes in patients with non-metastatic renal cell carcinoma (nmRCC) after curative nephrectomy during long-term follow-up. The medical records of 4,260 patients with nmRCC who underwent curative nephrectomy between 2000 and 2012 from five Korean institutions and follow-up after postoperative 1 month until December 2017 were retrospectively analyzed for RFS, MFS, OS, and CSS. During the median 43.86 months of follow-up, 342 recurrences, 127 metastases, and 361 deaths, including 222 cancer-specific deaths, were reported. In addition to the unreached median survival of RFS and MFS, the median OS and CSS times were 176.75 and 227.47 months, respectively. Multivariable analyses showed that nephrectomy type (laparoscopy vs. open), pathological T stages, and nuclear grade were common significant risk factors for survival, and the baseline ASA, hemoglobin, and pathological N stage were common factors only for RFS, OS, and CSS (*p* < 0.05). Further, tumor necrosis for MFS; platelet count, extent (partial vs. radical) of surgery, and lymphovascular invasion for RFS; baseline diabetes, hypertension, age, body mass index, extent of surgery, and pathological sarcomatoid differentiation for OS; and baseline diabetes, hypertension, body mass index, and pathological sarcomatoid differentiation for CSS were additionally significant risk factors (*p* < 0.05). RFS, MFS, OS, and CSS were significantly different depending on the pathological T stages (*p* < 0.05). In conclusion, this large-numbered, long-term follow-up study revealed significant factors affecting the survival of patients with nephrectomized nmRCC.

## Introduction

Worldwide, renal cell carcinoma (RCC) represents 2–3% of all cancers, with approximately 84,400 new patients and 34,700 kidney cancer-related deaths reported in the 2012 European Union Reports (1, 2). Advances in techniques have enabled earlier tumor detection and decreases in mortality over the last few decades (3). However, once recurrence or metastasis occurs, survival outcomes decrease to as low as <20–30% of the 5-year overall survival (OS) despite complete resection of localized or locally advanced RCC confined to the kidney (4–7).

Various prediction models including nomograms with significant prognostic factors are used to predict prognostic outcomes for future therapeutic application for patients with non-metastatic RCC (nmRCC) who have undergone curative surgery. Factors affecting the long-term outcomes of RCC after primary surgical resection depend on the characteristics of recurrent carcinoma or metastatic type, nephrectomy, and primary tumor, the treatment-free interval, or disease recurrence-free survival (RFS) (5–12). Nevertheless, recurrence or metastasis occurs even after 5 years of close follow-up with a 20–40% recurrence rate and a 5–15% metastasis rate depending on the pathological/clinical stages and tumor nuclear grade after curative nephrectomy (4, 5, 7–10).

Predicting survival, delayed recurrence, progression to metastasis, and aggressiveness in RCC despite complete resection of the primary kidney tumor is difficult because of the heterogeneity of RCC oncogenesis and presence of different tumor micro-environments regardless of the tumor size (5, 8, 10, 11). Additionally, limited long-term follow-up data following curative nephrectomy of primary kidney tumors are available. After 5 years of postoperative follow-up, patients are typically no longer followed. Therefore, in this study, data from over 4,000 patients with nmRCC who underwent nephrectomy were retrospectively collected from prospectively enrolled nephrectomized RCC cohorts from five tertiary institutions after long-term follow-up. This study was conducted to identify the prognostic risk factors of RFS, metastasis-free survival (MFS), OS, and cancer-specific survival (CSS) in patients with nmRCC after curative nephrectomy of the primary kidney tumor.

## Materials and Methods

### Ethics Statement

Following approval of this retrospective analytic study of the previously approved nephrectomized RCC database13 by the Institutional Review Board of the National Cancer Center (IRB No. NCC 2018-0045 and IRB number: B1202/145-102), the IRB approved exemption from written consent for this study. All study protocols were performed in accordance with the tenets of the ethical guidelines and regulations of the “World Medical Association Declaration of Helsinki-Ethical Principles for Medical Research Involving Human Subjects.”

### Patients' Criteria

Cohorts of patients with nephrectomized nmRCC were obtained from two previously existing Korean Nationwide Kidney Cancer databases from multiple Korean tertiary institutions. The first multicenter RCC database was a localized RCC database of 5,434 patients diagnosed with pT1-4NxM0 who underwent either partial or radical nephrectomy and were treated in seven Korean tertiary hospitals beginning in 2002 (National Cancer Center, Chonnam National University Hwasun Hospital, Seoul Saint Mary's Hospital, Seoul National University Hospital and Seoul National University Bundang Hospital) (13). The web-based metastatic RCC database created in 2015 was also used and included 6,849 patients with either synchronous or metachronous mRCC from 13 tertiary academic centers (Seoul National University Bundang Hospital, Seoul National University of Hospital, Seoul St. Mary's Hospital, Chungbuk National University College of Medicine, Cheongju, Korea, Department of Urology, Chonnam National University Hwasun Hospital, Kyungpook National University Medical Center, Korea University Medical Center, and National Cancer Center), treated between 1990 and 2015 and updated yearly until January 2018 (12).

From the localized RCC database (13) and metastatic RCC database (12), common patients between hospitals with recently updated follow-up were matched and registered patients from five hospitals (Chonnam National University Hospital, National Cancer Center, Seoul St. Mary's Hospital, Seoul National University Bundang Hospital, and Seoul National University Hospital) were selected after validating their survival outcomes according to national insurance numbers. A total of 5,434 patients with nmRCC who underwent either partial or radical nephrectomy for curative purpose with/without lymph node dissection between 2000 and 2012 and were followed-up until 2017 for at least 1 month of recurrence, metastasis, or death were selected. After excluding patients under 19 years old, those with benign tumor histology based on recent international genitourinary histopathology guidelines, those with a history of cytoreductive nephrectomy, and patients with incomplete medical records with respect to survival outcomes and analytic clinicopathological parameters, 4,260 patients were enrolled and analyzed for survival outcomes and predisposing risk factors.

The parameters analyzed in this study included baseline anthropometric and laboratory information regarding age, sex, comorbidities, albumin, hemoglobin, creatinine, American Society of Anethesiologists (ASA) score, nephrectomy information, and pathological information including pTNM stage, histology, Fuhrman nuclear grade, sarcomatoid differentiation, lymphovascular invasion, necrosis, and capsule invasion. Survival outcomes (RFS, MFS, OS, and CSS) were also obtained and analyzed. Operative information about surgical procedures of partial and radical nephrectomies was documented in a previously published paper (13); however, information regarding specific standardized protocol for surgical procedures was not included during data collection from the RCC database, as the data were collected retrospectively from multicenter databases.

### Statistical Analysis

Baseline characteristics are presented as frequencies (percentages) for categorical variables and mean ± standard deviation or median (interquartile range, IQR) for continuous variables. Differences in distributions were compared between metastatic and non-metastatic groups using *t*-test or Wilcoxon rank-sum test for continuous variables and Fisher's exact test or Pearson's chi-square test for categorical variables as appropriate. The loco-regional recurrence at operative field was diagnosed according to imaging studies. Recurrence occurring within 6 months from the operating date was defined as synchronous metastasis rather than postoperative recurrence. Therefore, RFS was defined as the time from nephrectomy to the diagnosis of loco-regional recurrence without metastasis during postoperative follow-up. MFS was defined as the time from nephrectomy to the diagnosis of metastasis during postoperative follow-up. OS was defined as all cause death after nephrectomy, and CSS was defined as only RCC-related death after nephrectomy.

Survival curves were estimated using the Kaplan-Meier method, and differences in RFS, MFS, OS, and CSS between groups were tested by log-rank test. The Cox proportional hazards model was used to evaluate the prognostic risk factors in RFS, MFS, OS, and CSS, and summarized as the hazard ratio (HR) and 95% confidence interval (CI). All results were considered statistically significant when two-sided *p*-values were < 0.05. Statistical analysis was performed using SAS 9.4 software (SAS Institute, Inc., Cary, NC, USA) and R software, version 3.5.0 (R Project for Statistical Computing).

## Results

### Baseline Characteristics

The mean age of the 4,260 patients at the time of surgery was 55.54 (standard deviation, 12.43) years. The male-to-female ratios and median follow-up duration (interquartile range) were 3,020/1,240 (70.9/29.1%) and 43.9 (19.0–76.1) months, respectively (Table 1). A total of 342 (8.1%) recurrences, 127 (3.0%) metastases, and 361 (8.5%) deaths including 222 (5.2%) RCC-related deaths were reported. The ratio of partial to radical nephrectomy and open to laparoscopy were 1,854/1,394 (43.5/32.7%), and 1,940/2,261 (45.5/53.1%), respectively.

**Table 1:** Baseline characteristics (*N* = 4,260).

		**Total**	**Non-metastasis**	**Metastasis**	***p*-value**
Number		4,260	4,133	127	
Follow-up duration	Median (IQR)	43.9 (19.0–76.1)	43.1 (18.6–74.3)	91.4 (39.4–122.3)	
Age at operation	Mean ± STD	55.5 ± 12.4	55.5 ± 12.5	57.6 ± 10.7	0.032
Gender	Male	3,020 (70.9)	2,929 (70.9)	91 (71.7)	0.848
	Female	1,240 (29.1)	1,204 (29.1)	36 (28.3)	
Body mass index (kg/cm^2^)	Mean ± STD	24.5 ± 3.4	24.5 ± 3.4	24.2 ± 2.9	0.232
Diabetes	Yes	602 (14.1)	581 (14.1)	21 (16.5)	0.432
Hypertension	Yes	1,602 (37.6)	1,545 (37.6)	57 (44.9)	0.095
ASA	1	1,419 (33.3)	1,385 (42.9)	34 (34.0)	0.301
	2	1,727 (40.5)	1,667 (51.6)	60 (60.0)	
	3	177 (4.3)	171 (5.3)	6 (6.0)	
	4	5 (0.1)	5 (0.2)	0 (0.0)	
Hb	Median (IQR)	13.9 (12.7–15.0)	13.9 (12.7–15.0)	13.8 (12.2–14.9)	0.131
Platelet	Median (IQR)	229 (193–271)	229 (192.5–270)	244 (194–292)	0.048
Creatinine	Median (IQR)	1.0 (0.8–1.1)	1.0 (0.8–1.1)	1 (0.9–1.2)	0.025
Albumin	Median (IQR)	4.4 (4.1–4.6)	4.4 (4.1–4.6)	4.2 (3.9–4.5)	<0.001
Nephrectomy	Open surgery	1,940 (45.5)	1,848 (45.3)	92 (76.0)	<0.001
	Laparoscopic	2,261 (53.1)	2,232 (54.7)	29 (24.0)	
Operative Extent	Partial	1,854 (43.5)	1,839 (57.6)	15 (26.3)	<0.001
	Radical	1,394 (32.7)	1,352 (42.4)	42 (73.7)	
pT	T1	3,397 (79.7)	3,353 (81.2)	44 (35.8)	<0.001
	T2	338 (7.9)	310 (7.5)	28 (22.8)	
	T3	490 (11.5)	442 (10.7)	48 (39.0)	
	T4	25 (0.6)	22 (0.5)	3 (2.4)	
	Tx	3 (0.1)	3 (0.1)	0 (0.0)	
pN	N0	1,784 (41.9)	1,696 (41.1)	88 (71.5)	<0.001
	N1	55 (1.3)	53 (1.3)	2 (1.6)	
	Nx	2,407 (56.5)	2,374 (57.6)	33 (26.8)	
Histology	Clear cell	3,589 (84.3)	3,476 (84.5)	113 (91.9)	0.077
	Non-clear cell	604 (14.2)	595 (14.5)	9 (7.3)	
	Mixed	45 (1.1)	44 (1.1)	1 (0.8)	
Nuclear grade	Grade 1–2	2,198 (51.6)	2,171 (55.2)	27 (25.7)	<0.001
	Grade 3–4	1,843 (43.3)	1,765 (44.8)	78 (74.3)	
Sarcomatoid differentiation	Yes	79 (1.9)	70 (1.7)	9 (7.1)	0.001
Necrosis	Yes	314 (7.4)	287 (6.9)	27 (21.3)	<0.001
Lymphovascular invasion	Yes	161 (3.8)	150 (3.6)	11 (8.7)	0.008
Capsular invasion	Yes	723 (17.0)	693 (16.8)	30 (23.6)	0.043
Cause of death (*n* = 361)	RCC related	222 (61.5)	184 (57.3)	38 (95.0)	<0.001
	Non-RCC-related	139 (38.5)	137 (42.7)	2 (5.0)	

The incidence of pathological T1-2/3-4/Tx and N1 stages were 3735/515/3(87.7/12.1/0.1%) and 55 (1.3%), respectively. The rate of histological types of clear cell/non-clear cell/unclassified were 3,589/604/67 (84.2/14.2/1.6%). The nuclear grades of 1–2/3–4/unknown were 2,198/1,843/219 (51.6/43.3/5.1%), respectively. There were detected 1.9% of sarcomatoid differentiation, 3.8% of tumor necrosis, 17.0% of lymphovascular invasion, and 17.0% of capsular invasion from the primary kidney tumor.

**Figure 1:**
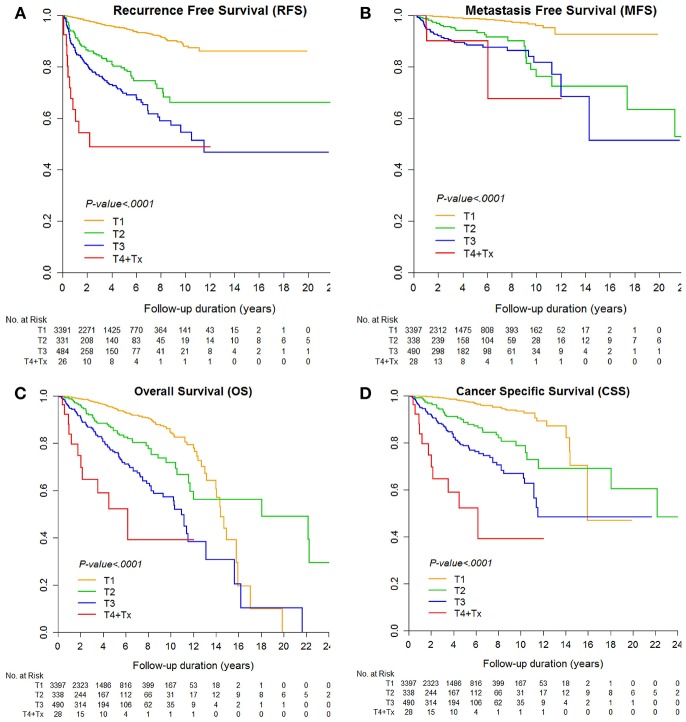
Kaplan-Meier curve for **(A)** recurrence-free survival, **(B)** metastasis-free survival, **(C)** overall survival, and **(D)** cancer-specific survival according to pathological T stages.

**Figure 2:**
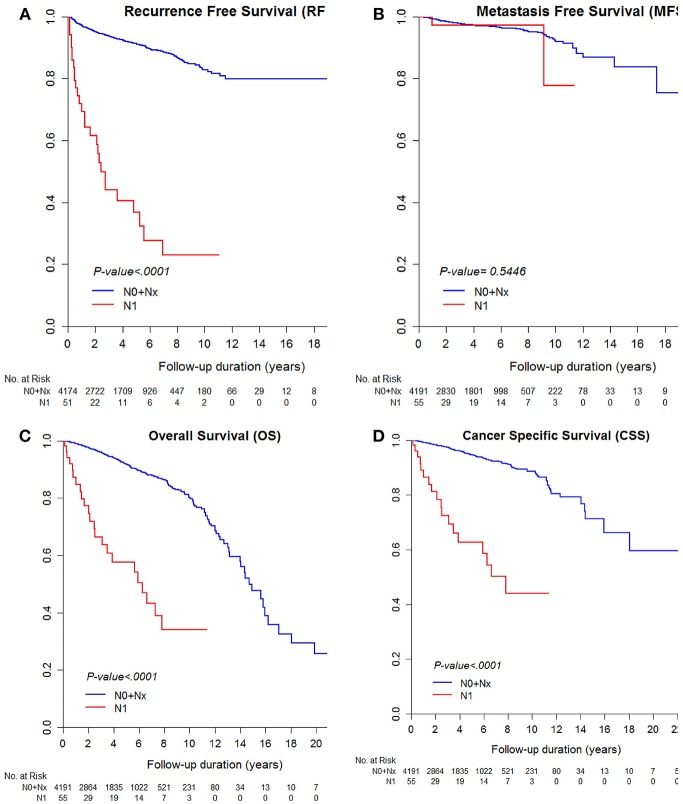
Kaplan-Meier curve for **(A)** recurrence-free survival, **(B)** metastasis-free survival, **(C)** overall survival, and **(D)** cancer-specific survival according to pathological N stages.

The median RFS and MFS were not reached, and median survival times were 176.8 and 227.5 months for OS and CSS (Table 2; Figures 1, 2). Different survival outcomes and their median times were analyzed according to pathological T and N stages when the MFS did not reach the median survival time in any T and N stages (Tables 1, 2; Figures 1, 2). Other baseline information including operative and pathological characteristics are summarized in Tables 1, 2.

**Table 2:** Median survival time according to pathological T stages and N stages.

		**Median survival time (95% CI)**			
		**RFS**	**MFS**	**OS**	**CSS**
Total (*n* = 4,260)		Not reached	Not reached	176.75 (159.58–194.70)	227.47 (208.31–inf)
Pathologic T stage	T1	Not reached	Not reached	172.67 (168.03–191.41)	191.41 (172.67–inf)
	T2	Not reached	Not reached	217.02 (140.38–inf)	266.14 (217.02–inf)
	T3	138.35 (106.19–inf)	Not reached	131.57 (111.78–157.32)	138.21 (133.87–inf)
	T4 + Tx	26.43 (6.87–inf)	Not reached	73.94 (24.53–inf)	73.94 (24.53–inf)
Pathologic N stage	N0 + Nx	Not reached	Not reached	176.75 (168.03–194.70)	266.14 (217.02–inf)
	N1	29.29 (14.99–63.26)	Not reached	75.49 (30.38–inf)	94.00 (41.98–inf)

### Multivariable Analysis for Metastasis-Free Survival Risk Factors

Multivariable analysis of predictive prognostic factors for MFS showed significant factors such as laparoscopic nephrectomy (HR 0.56, 95% CI 0.36–0.86), nuclear grade 3–4 (HR 2.78, 95% CI 1.75–4.40), presence of necrosis within the tumor (HR 1.72, 95% CI 1.08–2.75), and pathological T stage, in which increased pT stage had worse HRs (pT2/pT3/pT4, HR 3.55 [95% CI 2.15–5.87]/5.15 [95% CI 3.33–7.95]/5.20 [95% CI 1.56–17.37], comparing to pT1) (*p* < 0.05, Table 3A).

**Table 3:** Univariable and multivariable Cox proportional hazard model for **(A)** metastasis-free survival (MFS) and **(B)** recurrence-free survival (RFS).

**(A)**					
		**Metastasis-free survival (MFS)**			
		**Total = 4,260, events = 127**			
		**Univariable model**		**Multivariable model**	
		**HR (95% CI)**	***p*****-value**	**HR (95% CI)**	***p*****-value**
Age at operation		1.02 (1.01–1.04)	0.0072		
Body mass index (kg/cm^2^)		0.98 (0.92–1.03)	0.3861		
Diabetes	Yes	1.36 (0.85–2.17)	0.2048		
Hypertension	Yes	1.71 (1.20–2.44)	0.0031		
ASA	1 + 2	1 (ref)			
	3 + 4	1.33 (0.58–3.03)	0.502		
Hb	Female (≤ 12), male (≤ 13)	1 (ref)			
	Female (>12), male (>13)	0.52 (0.35–0.77)	0.0012		
Platelet	≥150, ≤ 450	1 (ref)			
	<150	0.90 (0.40–2.06)	0.8057		
	>450	5.29 (2.31–12.09)	<0.0001		
Creatinine	≤ 1.3	1 (ref)			
	>1.3	1.48 (0.83–2.64)	0.1824		
Albumin	≤ 3.0	1 (ref)			
	>3.0	0.77 (0.19–3.11)	0.7116		
Nephrectomy	Open surgery	1 (ref)		1 (ref)	
	Laparoscopic	0.38 (0.25–0.58)	<0.0001	0.56 (0.36–0.86)	0.008
Operative Extent	Partial	1 (ref)			
	Radical	2.66 (1.47–4.82)	0.0012		
pT	T1	1 (ref)		1 (ref)	
	T2	5.08 (3.13–8.25)	<0.0001	3.55 (2.15–5.87)	<0.0001
	T3	7.87 (5.22–11.86)	<0.0001	5.15 (3.33–7.95)	<0.0001
	T4 + Tx	11.25 (3.49–36.28)	<0.0001	5.20 (1.56–17.37)	0.0073
pN	N0 + Nx	1 (ref)			
	N1	1.53 (0.38–6.20)	0.5496		
Nuclear grade	Grade 1–2	1 (ref)		1 (ref)	
	Grade 3–4	4.35 (2.80–6.75)	<0.0001	2.78 (1.75–4.40)	<0.0001
Sarcomatoid differentiation	Yes	6.68 (3.38–13.2)	<0.0001		
Necrosis	Yes	3.87 (2.53–5.93)	<0.0001	1.72 (1.08–2.75)	0.0228
Lymphovascular invasion	Yes	2.35 (1.27–4.37)	0.0069		
Capsular invasion	Yes	1.38 (0.92–2.09)	0.1219		
**(B)**					
		**Recurrence-free survival (RFS)**			
		**Total = 4,239, events = 342**			
		**Univariable model**		**Multivariable model**	
		**HR (95% CI)**	***p*****-value**	**HR (95% CI)**	***p*****-value**
Age at operation		1.01 (1.00–1.02)	0.0216		
Body mass index (kg/cm^2^)		0.94 (0.91–0.98)	0.0013		
Diabetes	Yes	1.16 (0.86–1.57)	0.3203		
Hypertension	Yes	1.03 (0.83–1.29)	0.7789		
ASA	1 + 2	1 (ref)		1 (ref)	
	3 + 4	2.50 (1.62–3.85)	<0.0001	2.10 (1.35–3.26)	0.0009
Hb	Female (≤ 12), male (≤ 13)	1 (ref)		1 (ref)	
	Female (>12), male (>13)	0.36 (0.29–0.46)	<0.0001	0.61 (0.48–0.78)	<0.0001
Platelet	≥150, ≤ 450	1 (ref)		1 (ref)	
	<150	0.41 (0.19–0.86)	0.0189	0.38 (0.18–0.80)	0.0109
	>450	4.63 (2.65–8.09)	<0.0001	1.64 (0.92–2.94)	0.0941
**(A)**					
		**Metastasis-free survival (MFS)**			
		**Total = 4,260, events = 127**			
		**Univariable model**		**Multivariable model**	
		**HR (95% CI)**	***p*****-value**	**HR (95% CI)**	***p*****-value**
Creatinine	≤ 1.3	1 (ref)			
	>1.3	1.49 (1.04–2.13)	0.0292		
Albumin	≤ 3.0	1 (ref)			
	>3.0	0.42 (0.22–0.78)	0.0066		
Nephrectomy	Open surgery	1 (ref)		1 (ref)	
	Laparoscopic	0.42 (0.33–0.53)	<0.0001	0.72 (0.55–0.94)	0.0145
Operative Extent	Partial	1 (ref)		1 (ref)	
	Radical	3.99 (2.85–5.60)	<0.0001	1.79 (1.23–2.61)	0.0024
pT	T1	1 (ref)		1 (ref)	
	T2	4.39 (3.27–5.90)	<0.0001	2.03 (1.48–2.80)	<0.0001
	T3	6.93 (5.44–8.83)	<0.0001	3.24 (2.45–4.29)	<0.0001
	T4 + Tx	15.89 (8.82–28.64)	<0.0001	4.98 (2.63–9.43)	<0.0001
pN	N0 + Nx	1 (ref)		1 (ref)	
	N1	10.67 (7.25–15.72)	<0.0001	2.89 (1.90–4.40)	<0.0001
Nuclear grade	Grade 1–2	1 (ref)		1 (ref)	
	Grade 3–4	2.63 (2.03–3.42)	<0.0001	1.85 (1.41–2.44)	<0.0001
Sarcomatoid differentiation	Yes	3.57 (2.09–6.10)	<0.0001		
Necrosis	Yes	2.29 (1.69–3.12)	<0.0001		
Lymphovascular invasion	Yes	4.51 (3.30–6.18)	<0.0001	1.61 (1.12–2.32)	0.0097
Capsular invasion	Yes	1.47 (1.14–1.88)	0.0028		

### Multivariable Analysis for Recurrence-Free Survival Risk Factors

For significant factors of RFS, multivariable analysis showed ASA 3–4 (HR 2.1, 95% CI 1.35–3.26), no anemia (HR 0.61, 95% CI 0.48–0.78), thrombocytopenia (HR 0.38, 95% CI 0.18–0.80), laparoscopic nephrectomy (HR 0.72, 95% CI 0.55–0.94), radical nephrectomy (HR 1.79, 95% CI 1.23–2.61), pT stage (HR 2.03, 95% CI 1.48–2.80 for pT2; HR 3.24, 95% CI 2.45–4.29 for pT3; and HR 4.98, 95% CI 2.63–9.43 for pT4), pN1 stage (HR 2.89, 95% CI 1.90–4.40), nuclear grade 3–4 (HR 1.85, 95% CI 1.41–2.44), and presence of lymphovascular invasion (HR 1.61, 95% CI 1.12–2.32) (*p* < 0.05, Table 3B).

### Multivariable Analysis for Overall Survival and Cancer-Specific Survival Risk Factors

For OS and CSS, multivariable analyses showed common risk factors such as preoperative body mass index, diabetes, hypertension, and ASA 3–4 score, no anemia, nephrectomy type (laparoscopy vs. open), pT, pN stages, nuclear grade, and presence of sarcomatoid differentiation (*p* < 0.05, Table 4). Significant risk factors of OS were age (HR 1.03, 95% CI 1.02–1.04), preoperative body mass index (HR 0.93, 95% CI 0.89–0.97), diabetes (HR 1.73, 95% CI 1.30–2.29), hypertension (HR 1.39, 95% CI 1.09–1.79), ASA 3–4 score (HR 2.96, 95% CI 2.08–4.21), no anemia (HR 0.49, 95% CI 0.38–0.62), laparoscopic nephrectomy (HR 0.61, 95% CI 0.45–0.82), operative extent (partial vs. radical) (HR 1.50, 95% CI 1.02–2.20), pT3 stage (HR 1.98, 95% CI 1.50–2.60), and pT4 stage (HR 4.78, 95% CI 2.49–9.15), pathological N1 stage (HR 4.36, 95% CI 2.69–7.07), nuclear grade 3–4 (HR 1.77, 95% CI 1.33–2.35), and pathological sarcomatoid differentiation (HR 2.18, 95% CI 1.22–3.89) (*p* < 0.05, Table 4A).

**Table 4:** Univariable and multivariable Cox proportional hazard model for **(A)** overall survival (OS) and **(B)** cancer-specific survival (CSS).

**(A)**					
		**Overall survival (OS)**			
		**Total = 4,260, events = 361**			
		**Univariable model**		**Multivariable model**	
		**HR (95% CI)**	***p*****-value**	**HR (95% CI)**	***p*****-value**
Age at operation		1.04 (1.04–1.05)	<0.0001	1.03 (1.02–1.04)	<0.0001
Body mass index (kg/cm^2^)		0.92 (0.88–0.95)	<0.0001	0.93 (0.89–0.97)	0.0002
Diabetes	Yes	1.85 (1.44–2.39)	<0.0001	1.73 (1.30–2.29)	0.0001
Hypertension	Yes	1.40 (1.13–1.73)	0.0022	1.39 (1.09–1.79)	0.0093
ASA	1 + 2	1 (ref)		1 (ref)	
	3 + 4	4.18 (2.98–5.88)	<0.0001	2.96 (2.08–4.21)	<0.0001
Hb	Female (≤ 12), male (≤ 13)	1 (ref)		1 (ref)	
	Female (>12), male (>13)	0.27 (0.21–0.33)	<0.0001	0.49 (0.38–0.62)	<0.0001
Platelet	≥150, ≤ 450	1 (ref)			
	<150	1.30 (0.80–2.10)	0.2919		
	>450	3.56 (1.88–6.71)	<0.0001		
Creatinine	≤ 1.3	1 (ref)			
	>1.3	2.50 (1.86–3.37)	<0.0001		
Albumin	≤ 3.0	1 (ref)			
	>3.0	0.27 (0.16–0.48)	<0.0001		
Nephrectomy	Open surgery	1 (ref)		1 (ref)	
	Laparoscopic	0.31 (0.24–0.41)	<0.0001	0.61 (0.45–0.82)	0.0013
Operative Extent	Partial	1 (ref)		1 (ref)	
	Radical	2.82 (1.99–3.99)	<0.0001	1.50 (1.02–2.20)	0.0394
pT	T1	1 (ref)		1 (ref)	
	T2	1.97 (1.43–2.71)	<0.0001	1.06 (0.75–1.51)	0.7422
	T3	4.33 (3.42–5.49)	<0.0001	1.98 (1.50–2.60)	<0.0001
	T4 + Tx	10.46 (5.69–19.25)	<0.0001	4.78 (2.49–9.15)	<0.0001
pN	N0 + Nx	1 (ref)		1 (ref)	
	N1	6.52 (4.27–9.97)	<0.0001	4.36 (2.69–7.07)	<0.0001
Nuclear grade	Grade 1–2	1 (ref)		1 (ref)	
	Grade 3–4	2.34 (1.79–3.05)	<0.0001	1.77 (1.33–2.35)	0.0001
Sarcomatoid differentiation	Yes	3.79 (2.22–6.47)	<0.0001	2.18 (1.22–3.89)	0.0081
Necrosis	Yes	1.82 (1.32–2.53)	0.0003		
Lymphovascular invasion	Yes	2.66 (1.88–3.78)	<0.0001		
Capsular invasion	Yes	1.01 (0.78–1.32)	0.9257		
**(B)**					
		**Cancer-specific survival (CSS)**			
		**Total = 4,260, events = 222**			
		**Univariable model**		**Multivariable model**	
		**HR (95% CI)**	***p*****-value**	**HR (95% CI)**	***p*****-value**
Age at operation		1.03 (1.02–1.04)	<0.0001		
Body mass index (kg/cm^2^)		0.90 (0.86–0.94)	<0.0001	0.91 (0.86–0.95)	0.0001
Diabetes	Yes	1.83 (1.32–2.53)	0.0003	2.11 (1.47–3.02)	<0.0001
Hypertension	Yes	1.31 (1.00–1.72)	0.0533	1.48 (1.08–2.02)	0.0143
ASA	1 + 2	1 (ref)		1 (ref)	
	3 + 4	3.24 (2.00–5.26)	<0.0001	2.21 (1.34–3.64)	0.0019
Hb	Female (≤ 12), male (≤ 13)	1 (ref)		1 (ref)	
	Female (>12), male (>13)	0.24 (0.18–0.33)	<0.0001	0.46 (0.34–0.63)	<0.0001
Platelet	≥150, ≤ 450	1 (ref)			
	<150	0.65 (0.29–1.48)	0.3058		
	>450	3.76 (1.76–8.04)	0.0006		
**(B)**					
		**Cancer-specific survival (CSS)**			
		**Total = 4,260, events = 222**			
		**Univariable model**		**Multivariable model**	
		**HR (95% CI)**	***p*****-value**	**HR (95% CI)**	***p*****-value**
Creatinine	≤ 1.3	1 (ref)			
	>1.3	2.65 (1.83–3.86)	<0.0001		
Albumin	≤ 3.0	1 (ref)			
	>3.0	0.22 (0.12–0.43)	<0.0001		
Nephrectomy	Open surgery	1 (ref)		1 (ref)	
	Laparoscopic	0.29 (0.21–0.41)	<0.0001	0.61 (0.42–0.89)	0.0111
Operative Extent	Partial	1 (ref)			
	Radical	4.28 (2.57–7.12)	<0.0001		
pT	T1	1 (ref)		1 (ref)	
	T2	3.69 (2.50–5.46)	<0.0001	1.94 (1.27–2.96)	0.0022
	T3	7.76 (5.72–10.53)	<0.0001	3.58 (2.54–5.03)	<0.0001
	T4 + Tx	22.69 (12.06–42.69)	<0.0001	8.91 (4.52–17.56)	<0.0001
pN	N0 + Nx	1 (ref)		1 (ref)	
	N1	8.39 (5.17–13.62)	<0.0001	4.37 (2.57–7.42)	<0.0001
Nuclear grade	Grade 1–2	1 (ref)		1 (ref)	
	Grade 3–4	3.84 (2.66–5.54)	<0.0001	2.73 (1.83–4.07)	<0.0001
Sarcomatoid differentiation	Yes	5.57 (3.17–9.78)	<0.0001	2.22 (1.22–4.03)	0.0093
Necrosis	Yes	2.70 (1.89–3.86)	<0.0001		
Lymphovascular invasion	Yes	3.56 (2.39–5.30)	<0.0001		
Capsular invasion	Yes	1.21 (0.88–1.66)	0.2515		

For CSS, multivariable analysis showed preoperative body mass index (HR 0.91, 95% CI 0.86–0.95), diabetes (HR 2.11, 95% CI 1.47–3.02), hypertension (HR 1.48, 95% CI 1.08–2.02), ASA 3–4 score (HR 2.21, 95% CI 1.34–3.64), no anemia (HR 0.46, 95% CI 0.34–0.63), laparoscopic nephrectomy (HR 0.61, 95% CI 0.42–0.89), pT2 stage (HR 1.94, 95% CI 1.27–2.96), pT3 stage (HR 3.58, 95% CI 2.54–5.03) and pT4 (HR 8.91, 95% CI 4.52–17.56), pathological N1 stage (HR 4.37, 95% CI 2.57–7.42), nuclear grade 3–4 (HR 2.73, 95% CI 1.83–4.07), and pathological sarcomatoid differentiation (HR 2.22, 95% CI 1.22–4.03) as significant factors (*p* < 0.05, Table 4B).

## Discussion

Multiple predictive prognostic risk factors for nmRCC after curative surgery are considered as clinically important for aiding in patient counseling, determining optimal follow-up imaging protocols, and identifying patients who may benefit from early enrollment in adjuvant therapy protocols after surgery. This large, multicenter, retrospective study identified several significant risk factors of MFS, RFS, OS, and CSS and reported an 8.0% recurrence rate and 3.0% metastatic rate regardless of stage during a median follow-up of 44 months, similar to a previous study showing <5–10% of recurrence (7). Findings among the nmRCC cohort after complete removal of the primary kidney tumor for curative purpose are shown in Tables 3, 4.

We identified significant prognostic factors in common and similar prognostic hazard ratios of either favorable or unfavorable power for RFS, MFS, OS, and CSS (*p* < 0.05, Tables 2, 3). A favorable nephrectomy and unfavorable pT stage and nuclear grade in nmRCC have been already identified as potentially significant factors in many previous studies of prognosis prediction models in RCC (8–10, 14–19). The pathological T stage and Furhmann nuclear grade were suggested as the two most important prognostic factors representing the size and aggressive characteristics of the primary kidney tumor for clinicians in predicting a higher probability of delayed recurrence as well as progression to metastasis in nmRCC (9, 10, 15, 20, 21). In patients with a higher nuclear grade 3–4 and pathological presence of necrosis within the primary tumor, a higher rate of aggressive and rapidly growing metastatic tumor either hematogenously or via lymphatic spread to distant sites resulted in shorter MFS (Table 3) (16, 17, 21).

In addition to pT and nuclear grade, tumor characteristics including pathological N1 stage, presence of either sarcomatoid differentiation or lymphovascular invasion, and presence of underlying hypertension and diabetes significantly influence recurrence or mortality (18, 22). In contrast, some factors favor the prevention of tumor recurrence or mortality after surgery. A good performance status such as a higher level of baseline hemoglobin, platelet, and body mass index reflects not only increased immunity for killing tumor cells and defending against recurrence, but also increased tolerability of systemic or focal therapy for recurrent tumors because of better nutritional states, resulting in significantly lower recurrence and mortality. Similarly, Ahmedov et al. found a significant prognostic significance for body mass index, surgical type (partial/radical), and tumor stage for CSS and RFS in patients with localized, non-metastatic, unilateral RCC who underwent curative nephrectomy (23).

Age and the extent of nephrectomy were only significant factors for OS, but not for CSS (*p* < 0.05, Table 4). Other parameters showed similar values and hazard ratios. Thus, the mortality of patients with nmRCC who underwent curative nephrectomy depended upon senescence during aging and renal functional preservation after surgery (18, 19, 23). The nephron-sparing technique should be considered first for patients with nmRCC for curative nephrectomy of primary kidney tumor, reducing the nephron injury during surgery, and following patients in a nephrology clinic postoperatively to prevent chronic kidney disease. In addition, radical nephrectomy was an unfavorable risk factor because RCC with large tumor, hilar location, and more locally advanced tumor (local invasion, lymph nodal enlargement, intravena caval thrombi had a tendency to undergo radical nephrectomy resulting in poorer prognoses (17, 20, 22, 24).

Interestingly, RFS, OS, and CSS shared several common significant prognostic factors, such as ASA, hemoglobin, and pN stage, whereas MFS did not (*p* < 0.05, Tables 2, 3). This may provide clues for differentiating the different characteristics of influential parameters between metastasis and recurrence in nmRCC after curative surgery. Distinct parameters between MFS compared to RFS, OS, and CSS are important for developing prediction models of RFS and MFS. Significant prognostic factors for RFS, OS, and CSS showed many similarities in composition and hazard ratios for each parameter, unlike those for MFS. Further analytic study is needed to determine whether the recurrent RCC impacted more strongly on the OS and CSS in nmRCC that underwent curative nephrectomy, compared to metastatic RCC.

Recurrent RCC exhibited tumor characteristics similar to previous primary RCC that was surgically removed and affected survival outcomes (25). Recurrent RCC had already adapted to the microenvironment of the surgical bed that the progression to metastasis resulting in cancer death was much more easily processed than mRCC. In contrast, heterogenous mRCC may have more time to adapt to the new metastatic microenvironment and escape from the immune defense system, resulting in a greater influence on mortality compared to recurrent RCC. We performed a multiple sequential series of mRCC studies to define the characteristics of primary RCC and mRCC by tissue microarray, immunohistochemistry, and genetic analyses (11, 26). We found that less than half of metastatic tumors possessed similar mutations to the primary tumor, with most mRCC cases showing sequence differences from primary RCC, which allowed the cells to evade the immune system and undergo metastasis (26).

mRCC uses different metastatic pathways such as lymphatic or hematogenous spread compared to direct invasion of recurrent RCC and lymphatic and hematogenous spread leading to OS and CSS (10, 12). Another explanation of close relativity of recurrent RCC with OS and CSS might be no postoperative presence of successful adjuvant therapies established in the present era although some clinical trials have been going on the efficacy of the adjuvant target agents for the postoperative nmRCC underwent nephrectomy (4, 7, 13). More efficient adjuvant therapies are needed to treat high-risk nmRCC. Additionally, systemic therapy, such as antiangiogenic-targeted therapy and immune therapy, may more effectively control the metastatic tumor microenvironment following complete surgical removal to decrease the tumor burden (4, 5, 8), whereas recurrent RCC would be treated by focal intervention such as radiation therapy, cryotherapy, or surgery as early as possible.

Accordingly, mRCC should be considered a different heterogenic RCC from primary kidney tumor, that spreads through systemic circulation. Recurrent RCC should be considered as a progression of the primary kidney tumor, which is correlated more significantly with OS and CSS. This explains why baseline parameters before surgery were significant factors in RFS, OS and CSS; significant parameters for MFS were more related to the aggressive characteristics and tumor burdens of the primary kidney tumor rather than the patients' anthropometric states (14).

Finally, the current 5 year postoperative follow-up guideline should be greatly extended. Even in subgroup cohorts of pT1-2 stages, local recurrence and metastasis were 4.9% (*N* = 211) and 1.7% (*N* = 72), which agrees with previous active surveillance study showing a 1.4–6.7% late metastatic rate in <4 cm small RCC (15). These patients showed recurrence even after postoperative 5 year follow-up without reaching the median RFS and MFS until 263.7 months of follow-up (Table 2, Figures 1A,B). Another recent probability assessment study showed that tumor aggressiveness exhibited proportional increases of 18%, 24%, and 29% in the likelihood of aggressive histology for 2, 3, and 4 cm RCCs (27). A different study suggested that routine follow-up periods for earlier detection of metastasis or recurrence until 12 years would enable timely interventive treatment to improve survival outcomes, particularly in cases of small tumor burden (28). Patients lost to follow-up reported poor prognostic outcomes (29) and greater tumor burdens, and more progressive metastatic states showed higher tumor heterogeneity in RCC (16). Analysis of the predisposing risk factors identified in this study for postoperative recurrence or metastasis may enable better control of high-risk recurrence and metastasis to improve survival in RCC (Figures 1C,D).

This study has some limitations. The study used a retrospective, multicenter design although data were prospectively collected; selection bias may have occurred for non-standardized surgical protocols, which depended on the surgeon's discretion, and the absence of a central pathological data review. Moreover, recurrence and metastatic rates may have increased for longer follow-up durations. However, this is the first, large-scale, multicenter study to suggest multiple significant prognostic risk factors for RFS, MFS, OS, and CSS and the necessity of long-term surveillance protocols among patients with nmRCC who underwent nephrectomy.

## Conclusion

This large cohort study revealed multiple significant prognostic factors of various survival outcomes and addressed difficulties in analyzing prognostic predictive factors affecting survival in a surgical cohort with a low rate of recurrence or metastasis after long-term follow-up. These factors may be useful for predicting survival outcomes and enable the validation of previously identified prognostic factors.

## Data Availability

The datasets analyzed in this manuscript are not publicly available. Requests to access the datasets should be directed to cjs5225@ncc.re.kr or irb@ncc.re.kr.

## Author Contributions

SK and CJ: conceptualization, data curation, investigation, methodology, project administration, supervision, and writing—original draft preparation. EH, S-HH, CJ, CK, and SB: conceptualization, data curation, investigation, methodology, supervision, and writing—original draft preparation. BP: conceptualization, data curation, formal analysis, investigation, methodology, project administration, supervision, and writing—original draft preparation. JC: conceptualization, data curation, investigation, methodology, project administration, supervision, funding acquisition, and writing—original draft preparation.

### Conflict of Interest Statement

The authors declare that the research was conducted in the absence of any commercial or financial relationships that could be construed as a potential conflict of interest.
